# Supplementary material for: Low Serological Agreement of Hepatitis E in Immunocompromised Cancer Patients: A Comparative Study of Three Anti-HEV Assays
Source: Antibodies (Basel). 2025 Mar 24;14(2):27. doi: 10.3390/antib14020027 (PMC12015928; doi:10.3390/antib14020027)
Supplement: Supplementary file 1 [file antibodies-14-00027-s001.zip › antibodies-3469328-supplementary.pdf]

Table S1 Questionnaire

Adapted Patient Questionnaire

Risk Factors of Hepatitis E Infection
Version 1.3, 01.07.2020

Based on PD Dr. Pischke, Dr. Horvatits

QUESTIONNAIRE

Study ID: ____________
Date: ______________
Gender (m/f): _____
Age (in years):
☐ < 30 ☐ 30-55 ☐ >55

ALL INFORMATION IS VOLUNTARY AND ANONYMOUS
INDIVIDUAL QUESTIONS MAY BE LEFT UNANSWERED

1. Do you work in agriculture without livestock (fruit/vegetable farming)?
☐ Yes ☐ No

2. Do you work professionally with wastewater (plumber/sewage treatment plant/city sanitation)?
☐ Yes ☐ No

3. How often do you eat cooked/fried pork or pork products?
☐ Never ☐ 1-5 times per year ☐ 6-12 times per year ☐ More than once per month

4. How often do you eat raw/undercooked pork products (e.g., minced pork/tartare)?
☐ Never ☐ 1-5 times per year ☐ 6-12 times per year ☐ More than once per month

5. How often do you eat game meat?
☐ Never ☐ 1-5 times per year ☐ 6-12 times per year ☐ More than once per month

6. How often do you eat spinach or arugula?
☐ Never ☐ 1-5 times per month ☐ 6-12 times per month ☐ Daily

7. Do you drink alcohol?
☐ Yes ☐ No
If yes:
☐ Daily (Amount: ___________)
☐ Once per week (Amount: ___________)
☐ Less than once per week (Amount: ___________)
